# Povidone iodine irritant dermatitis

**DOI:** 10.4103/0253-7613.62404

**Published:** 2010-02

**Authors:** Viroj Wiwanitkit

**Affiliations:** Wiwanitkit House, Bangkhae, Bangkok Thailand 10160. E-mail: wviroj@yahoo.com

I read the publication by Murthy *et al*., with a great interest.[1] They reported a case of povidone iodine-induced irritant dermatitis.[1] Indeed, povidone iodine-induced dermatitis is not extremely rare. There are several reports of irritant and allergic dermatitis due to povidone iodine. Severe anaphylaxis due to povidone iodine is also mentioned in literature.[2] Indeed, the differential diagnosis between both conditions is sometimes difficult and needs specific allergic tests.[3] However, in this case report,[3] Murthy *et al*., did not reach this step. In addition, it should be noted that the irritant condition due to povidone iodine usually relates to an outdated solution.[4] The data on the expired date of povidone iodine should be assessed in every case of povidone iodine dermatitis.
